# Electrochemical conversion of methane to ethylene in a solid oxide electrolyzer

**DOI:** 10.1038/s41467-019-09083-3

**Published:** 2019-03-12

**Authors:** Changli Zhu, Shisheng Hou, Xiuli Hu, Jinhai Lu, Fanglin Chen, Kui Xie

**Affiliations:** 1 Key Laboratory of Design and Assembly of Functional Nanostructures, Fujian Institute of Research on the Structure of Matter, Chinese Academy of Sciences, 350002 Fuzhou Fujian, China; 2 Department of Mechanical Engineering, University of South Carolina, 300 Main Street, Columbia, SC 29208 USA

## Abstract

Conversion of methane to ethylene with high yield remains a fundamental challenge due to the low ethylene selectivity, severe carbon deposition and instability of catalysts. Here we demonstrate a conceptually different process of in situ electrochemical oxidation of methane to ethylene in a solid oxide electrolyzer under ambient pressure at 850 °C. The porous electrode scaffold with an in situ-grown metal/oxide interface enhances coking resistance and catalyst stability at high temperatures. The highest C_2_ product selectivity of 81.2% together with the highest C_2_ product concentration of 16.7% in output gas (12.1% ethylene and 4.6% ethane) is achieved while the methane conversion reaches as high as 41% in the initial pass. This strategy provides an optimal performance with no obvious degradation being observed after 100 h of high temperature operation and 10 redox cycles, suggesting a reliable electrochemical process for conversion of methane into valuable chemicals.

## Abstract (web summary)

Conversion of methane into ethylene is important for chemical feedstocks, but is challenged by catalysts with low selectivity and performance degradation. Here the authors report electrochemical oxidation of methane in a solid oxide electrolyzer, achieving ethylene production with high selectivity and yield.

## Introduction

Conversion of natural gas, mainly CH_4_, into transportable fuels and chemicals has been largely driven for several decades by emerging industrial trends, including the rising demand for H_2_ to upgrade lower-quality oils and a global shortage of aromatics caused by shifting refinery targets toward gasoline^1^. Light olefins, mainly C_2_H_4_, as key chemical feedstocks and building blocks, are currently produced through multistage processes from syngas to methanol and then to olefins, although there is some ongoing research of direct conversion of syngas to olefins. This syngas-to-olefin route is dominant in current and near-term industry production^2–5^; however, the carbon-atom efficiency is normally below 50% in addition to the significant amount of CO_2_ emission. This mainly arises since the removal of oxygen in carbon monoxide consumes hydrogen and generates water.

Direct conversion of CH_4_ to C_2_H_4_ is potentially more economical and environmental friendly, but it is a more challenging route. CH_4_ is a small and stable molecule that exists in nature, and it has strong C–H bonds, negligible electron affinity, large ionization energy, and low polarizability. This direct-conversion route can be realized through either oxidative coupling or direct nonoxidative conversion in a heterogeneous catalysis process^1^. It is effective to activate the C–H bond of CH_4_ with oxygen to generate C_2_H_4_; however, over-oxidation of CH_4_ remains unavoidable, leading to lower carbon atom efficiency. Furthermore, the low CH_4_ conversion with low selectivity of C_2_H_4_ is another huge challenge^6,7^. Nonoxidative conversion is a process of dehydrogenation of highly stable CH_4_ together with selective C–C coupling, and a recent work has represented the highest methane conversion (48.1%) and ethylene selectivity (48.4%) at 1090 °C ^8^. In terms of reaction mechanism, the cleavage of a C–H bond with C–C coupling is indeed an oxidation process for either oxidative coupling or nonoxidative conversion of CH_4_ according to the charge balance of CH_4_ → C_2_H_4_ transformation. This process would be ideally realized using a conceptually different approach in the form of an efficient electrochemical process with a reaction $(2{\mathrm{CH}}_4 + {\mathrm{O}}^{2 - } \leftrightarrows {\mathrm{C}}_2{\mathrm{H}}_4 +{\mathrm{H}}_2 +{\mathrm{H}}_2{\mathrm{O}} + 2{\mathrm{e}}^ - )$ for the oxidative coupling of CH_4_ to C_2_H_4_ or with a reaction $(2{\mathrm{CH}}_4 - 4{\mathrm{e}}^ - \leftrightarrows {\mathrm{C}}_2{\mathrm{H}}_4 + 4{\mathrm{H}}^ + )$ for the nonoxidative conversion of CH_4_ to C_2_H_4_ through a synergistic control of electrochemical oxidation with in situ catalysis on the electrodes.

**Fig. 1:**
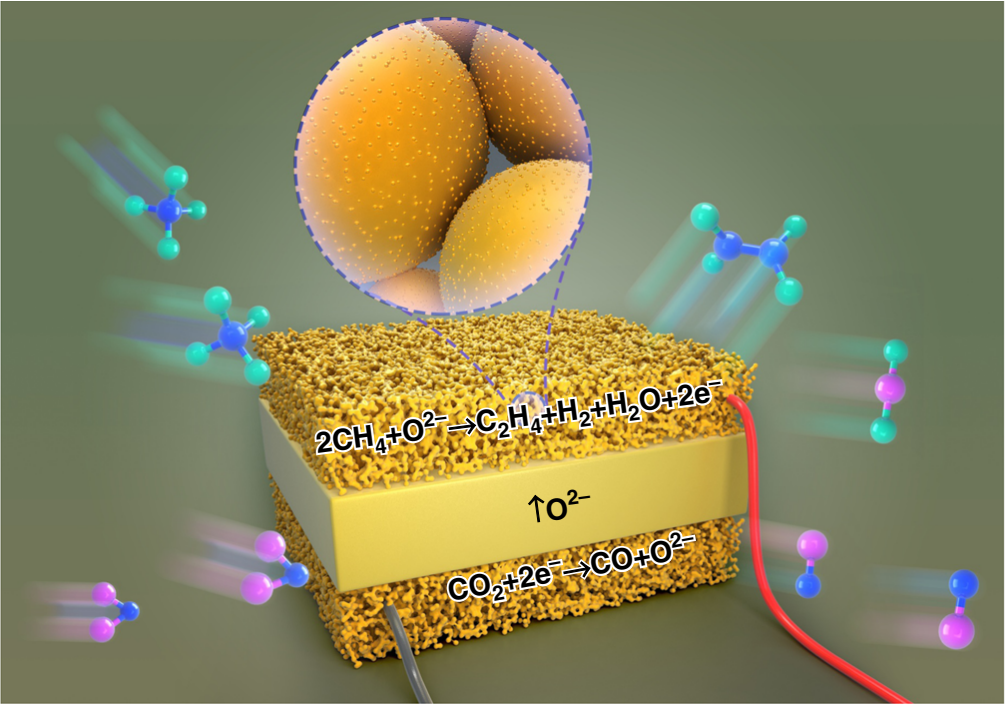
Electrochemical oxidation of CH_4_ to chemicals. Here the CO_2_ is converted to CO in the cathode while CH_4_ is converted to chemicals, such as C_2_H_4_ in the anode through a simultaneous process in a solid oxide electrolyzer

Solid oxide electrolyzers (SOEs) have been attracting great interest because of their high efficiencies in converting electricity into fuels and chemicals using renewable electrical energy^9^. The advantages, including long life, versatile scale, and low cost, have also demonstrated huge practical application potentials. They can exploit available high-temperature exhaust heat stream to maximize the electrical energy efficiency, and the high operation temperature of around 800–900 ^o^C well fits the requirement of catalytic conversion of CH_4_ to C_2_H_4_^1,10–14^. In an oxide-conducting SOE, the oxygen ions are transported from the cathode through the electrolyte membrane to the anode and form oxygen gas $\left( {{\mathrm{O}}^{2 - } - 2{\mathrm{e}}^ - \leftrightarrows 1/2{\mathrm{O}}_2} \right)$, whilst the cathode would provide suitable sites to accommodate electrolysis reaction that electrochemically extracts O^2–^ ions from gaseous molecules that contain oxygen atoms under an externally applied potential. As shown in Fig. 1, this unique mechanism provides a possibility of direct utilization of O^2−^ ions to in situ electrochemically oxidize CH_4_ $(2{\mathrm{CH}}_4 + 2{\mathrm{O}}^{2 - } \leftrightarrows {\mathrm{C}}_2{\mathrm{H}}_4 + 2{\mathrm{H}}_2{\mathrm{O}} + 4{\mathrm{e}}^ - )$ into C_2_H_4_ in the anode. In this case, the electrochemical conversion of CH_4_ into C_2_H_4_ would be successfully achieved, demonstrating economic and sustainable potential if this process exploits available exhaust heat stream and renewable electrical energy. Similar conversion of CH_4_ into C_2_H_4_ $(2{\mathrm{CH}}_4 - 4{\mathrm{e}}^ - \leftrightarrows {\mathrm{C}}_2{\mathrm{H}}_4 + 4{\mathrm{H}}^ + )$ would also be feasible in proton-conducting solid oxide electrolyzer while CH_4_ cracking might lead to coke formation in the electrode.

Electrochemical oxidation of CH_4_ to C_2_H_4_ in an oxide-ion conducting solid oxide electrolyzer involves the key step of the first cleavage of C–H bonding in CH_4_ and then the selective coupling of C–C bonding for C_2_H_4_ generation through a gaseous electrochemical process in the porous anode. The electrochemical pumping of O^2−^ ions from the cathode to the anode is indeed a process of generating active oxygen species for CH_4_ activation and oxidation in the presence of suitable catalysts. Electrochemical oxidation of CH_4_ with O^2−^ ions is also a charge-rebalancing process while the transformation of O^2−^ ions to the active interfaces in the anode would involve different types of oxygen species including O^2−^ and O^−^ ions to initiate the CH_4_ oxidation process^15–19^. Active metal–oxide interfaces at nanoscale would be ideal to accommodate these oxygen species especially in the form of O^−^ ions. These active oxygen species are one kind of the key active radicals at the confined metal–oxide interfaces to activate CH_4_ while the electrochemical pumping of O^2−^ ions would continuously oxidize CH_4_ in conjunction with gaseous selective coupling to generate C_2_H_4_ in the presence of suitable interface catalysis. Electrochemical pumping of active oxygen species to the porous anode would facilitate the oxidative removal of deposited carbon on the electrode scaffold and therefore enhance the coking resistance during the CH_4_ cracking process. The applied potentials would split the oxygen-containing molecules such as gaseous O_2_, H_2_O, or CO_2_ in the cathode to generate O^2−^ and simultaneously transport them to the anode. This feature provides the unique advantage of directly electrolyzing H_2_O or CO_2_ to H_2_ or CO to generate oxygen species in conjunction with selective oxidation of CH_4_ to C_2_H_4_ in the anode.

The incorporation of metal nanoparticles on oxide scaffold has been widely proven to be quite effective to enhance catalytic conversion of CH_4_ to C_2_H_4_ and the typical metal nanocatalysts, such as iron nanoparticles are normally considered to be suitable to construct metal–oxide interfaces with strong interactions to facilitate the catalysis conversion^20,21^. These metal–oxide interfaces are usually prepared by directly loading metal nanoparticles on porous substrates through an impregnation method. However, long-term stability of metallic nanoparticles simply loaded on oxide surface remains a major challenge. The severe agglomeration and sintering of these nanoparticles lead to catalysis performance degradation especially at high temperature. An alternative method is to dope the iron element in the host lattice of oxide scaffold during the catalyst synthesis in air and then in situ grow the metallic nanoparticles on oxide surface through a phase decomposition process under reducing conditions^10,14,22–24^. The metal nanoparticles directly embedded on porous oxide backbones particularly exhibit enhanced high-temperature stability and coking resistance, which may be due to the strong metal–oxide interactions at the unique interface architectures at nanoscale. Strong interface interactions are also highly favorable for the transfer of oxygen species especially in the form of oxygen ions in this work. These active oxygen species are expected to in situ electrochemically activate and oxidize CH_4_, and even remove the deposited carbon if present at metal–oxide interfaces in the cathode.

Here, we utilize redox-reversible layered perovskite Sr_2_Fe_1.5_Mo_0.5_O_6−*δ*_ (SFMO) anode to electrochemically oxidize CH_4_ to C_2_H_4_ in an oxide-ion-conducting solid oxide electrolyzer. Ceramic SFMO is a mixed conductor with rich oxygen vacancies and we have already shown its excellent activity and coking resistance toward CH_4_ oxidation^25–27^. We firstly in situ construct metal–oxide interfaces at nanoscale on porous SFMO scaffold by synergistic control of nonstoichiometry and doping of Sr_2_Fe_1.5+*x*_Mo_0.5_O_6−*δ*_ (*x* = 0–0.1). We then tailor the amount of excess iron to grow iron nanoparticles with different metal nanoparticle distribution on the SFMO scaffold through a phase decomposition process in reducing atmospheres. We then investigate the electrochemical pumping of oxygen species from air in the cathode to the anode for the selective electro-oxidation of CH_4_ to C_2_H_4_. We finally study the electrochemical oxidation of CH_4_ to chemicals in the anode in conjunction with electrolysis of CO_2_ to CO in the cathode.

## Results

### Growth of exsolved interfaces

**Fig. 2:**
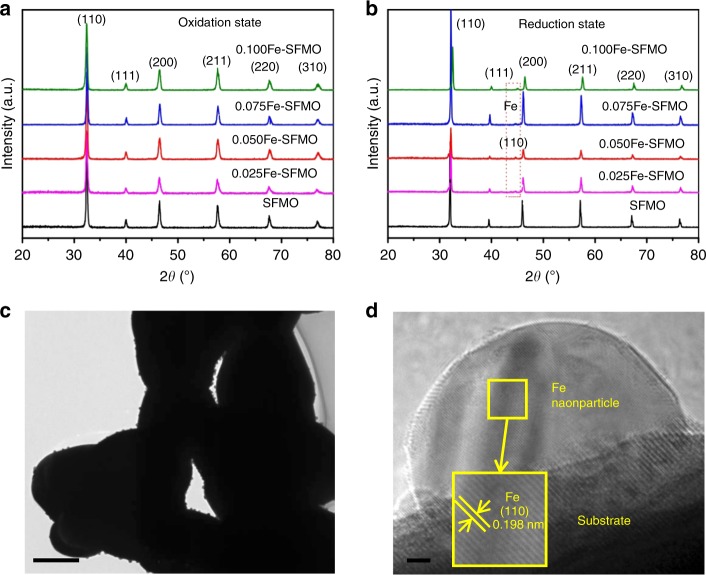
X-ray diffraction patterns and micrographs. **a** X-ray diffraction (XRD) patterns of oxidized samples. **b** XRD patterns of reduced samples. **c** High-resolution transmission electron microscopy (HRTEM) view of iron nanoparticles and the scale bar is 500 nm. **d** Enlarged view of iron nanoparticle anchored on the SFMO surface after reduction and the scale bar is 2 nm

Figure 2a shows the X-ray diffraction (XRD) patterns of the oxidized Sr_2_Fe_1.5+*x*_Mo_0.5_O_6−*δ*_ (*x* = 0–0.1) samples while the excess iron element is doped into the oxide lattice to form a series of homogeneous solid solutions. The chemical formulas of Sr_2_Fe_1.5_Mo_0.5_O_6−*δ*_, Sr_2_Fe_1.525_Mo_0.5_O_6-δ_, Sr_2_Fe_1.55_Mo_0.5_O_6-*δ*_, Sr_2_Fe_1.575_Mo_0.5_O_6-*δ*_, and Sr_2_Fe_1.6_Mo_0.5_O_6−*δ*_ are denoted as SFMO, 0.025Fe–SFMO, 0.050Fe–SFMO, 0.075Fe–SFMO, and 0.100Fe–SFMO, respectively. The presence of excess iron in the host oxide lattice would result in metallic iron nanoparticles on the SFMO scaffold after reduction, producing the electrochemically active metal–oxide interfaces through a phase decomposition process. The iron in the host lattice is mainly present in the form of Fe^3+/4+^ oxidation state as shown in the X-ray photoelectron spectroscopy (XPS) in Supplementary Fig. 1. The oxidized sample has a porous microstructure with clean scaffold surface as shown in Supplementary Fig. 2, and the lattice spacing of 0.288 nm in the transmission electron microscopy (TEM) is in accordance with (002) plane. Figure 2b shows the XRD of Sr_2_Fe_1.5+*x*_Mo_0.5_O_6−*δ*_ (*x* = 0–0.1) after reduction, confirming the presence of metallic iron phase that arises from the exsolution of excess iron dopant in the oxide lattice upon reduction. We further determine the oxygen nonstoichiometry of Sr_2_Fe_1.5+*x*_Mo_0.5_O_6−*δ*_ (*x* = 0–0.1) before and after reduction using iodometric titration and summarize the results in Supplementary Table 1. For the SFMO sample, the oxygen storage capacity is 0.2465 mol after reduction, indicating that the reversible oxygen loss may be mainly from the valence change of Fe/Mo in the lattice, while no iron nanoparticle exsolution is observed in this process. After reduction, the excess iron (*x* = 0.025–0.1) has been transformed into metallic iron nanoparticles while the left iron remains unchanged in a fully oxidized state. The exsolution of iron nanoparticles further leads to oxygen loss which shows a strong dependence of oxygen nonstoichiometry on the exsolution of iron metal as shown in Supplementary Table 1, which again validates the exsolution of up to 95–100% excess iron from the oxide lattice through a phase decomposition process during reduction. Figure 2c shows the uniform metallic iron nanoparticles with diameters of ~25 nm anchoring on the SFMO oxide surface after reduction in hydrogen atmosphere, which confirms the in situ growth of metal–oxide interfaces through a phase decomposition process. Figure 2d shows the in situ grown metal–oxide interfaces with the iron nanoparticles deeply embedded on the SFMO substrate, which demonstrates a clear heterojunction interface that is anticipated to produce strong interfacial interaction. At high temperature, the strong interface interactions at nanoscale would provide enhanced thermal stability against nanoparticle agglomerations and carbon deposition from CH_4_ pyrolysis.

**Fig. 3:**
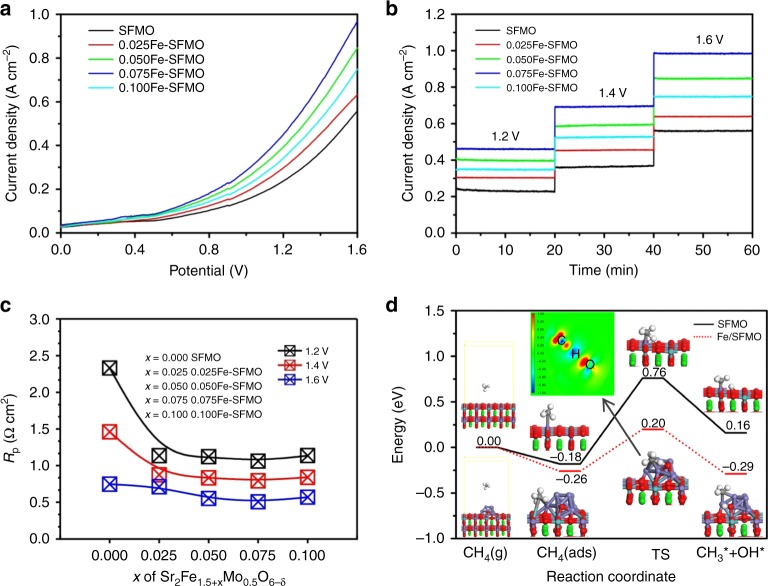
Electrochemical oxidation of CH_4_ at the anode in conjunction with O_2_ electrolysis. **a** The current density–voltage (*I–V*) curves of the electrolyzers with different cathodes for CH_4_ oxidation at 850 °C. **b** The short-term operation of CH_4_ oxidation with the composite anodes at different voltages. **c** The electrode polarizations with different cathodes at 1.2–1.6 V at 850 °C. **d** Energy diagrams for adsorption and C–H bond cleavage of CH_4_ on SFMO (001) and Fe/SFMO (001) including their initial, transition, and final states. Inset: electronic charge density difference for the transition state of Fe/SFMO (001)

SOEs with porous SFMO anodes are constructed while the cell microstructures are shown in Supplementary Fig. 3. We use La_0.9_Sr_0.1_Ga_0.8_Mg_0.2_O_3−*δ*_ (LSGM) electrolyte with dimension of 1 mm in thickness and 20 mm in diameter. LSGM is an excellent oxygen-ion-conducting electrolyte with high ionic conductivity at intermediate to high temperatures and we conduct CH_4_ oxidation at 850 °C to enhance the C_2_H_4_ yield. The electrochemical oxidation of CH_4_ in the anode is conducted in conjunction with O_2_ electrolysis from air in the cathode at 850 °C. The CH_4_/O_2_ fuel cell mode is a mode of electrochemical oxidation of CH_4_ with O_2_ gas toward CO_2_ generation driven by the oxygen gradient across two different electrodes in a concentration cell. Here we further apply voltages to boost the electrochemical CH_4_ oxidation process toward C_2_H_4_ generation in the presence of interface catalysis in the anode. Before operation, the anodes are pre-reduced using 5%H_2_/Ar to in situ grow the exsolved interfaces that are composed of iron nanoparticles anchoring on the SFMO scaffolds. Supplementary Fig. 4 shows that the iron nanoparticles are grown on the SFMO scaffold and the size of the iron nanoparticles has a narrow distribution with a mean diameter of ~25 nm after pre-reduction. Figure 3a shows that the current–voltage (*I*–*V*) relationship clearly reveals the superior performance with up to 100% enhancement of the SFMO cathodes with nanoscale metal–oxide interfaces in comparison to the bare cathode. The optimum chemical composition of the anode is found to be 0.075Fe–SFMO. The growth of metal–oxide interfaces drastically improves the current densities to ~1 A cm^−2^ at 1.6 V with the presence of optimum length of metal–oxide interfaces in the porous SFMO scaffolds. Supplementary Fig. 5 shows that the electrode polarization resistance is as low as ~0.4 Ω cm^2^ for the different anodes at the applied voltages of 1.6 V, indicating the significantly enhanced electrode process at higher applied voltages. Figure 3b shows the short-term operation of CH_4_ oxidation at different voltages, which again confirms the significantly enhanced electrochemical process with metal–oxide interfaces in the anodes. Figure 3c summarizes the electrode polarizations of the electrochemical oxidation of CH_4_ with different cathodes at 1.2–1.6 V at 850 °C. It is observed that the reduction of electrode polarization resistances strongly depends on the growth of metal–oxide interfaces in the electrode while higher applied voltage facilitates the electrochemical process and therefore enhances the electrode reaction kinetics. It should be noted that the overall electrode activity is enhanced with the growth of metal–oxide interfaces; however, more attentions need to be paid to the conversion of CH_4_ to C_2_H_4_ with less over-oxidation of CH_4_ to CO_2_ in the porous anodes.

### Methane conversion at interfaces

The properties of CH_4_ explain the exceptionally high molecule stability which therefore leads to the difficulties of CH_4_ activation and conversion. A limitation with most existing heterogeneous catalysts is that the initial C–H bond cleavage is the rate controlling step. It is widely accepted that the oxidation reaction of CH_4_ to C_2_H_4_ is usually initiated on the surface of solid catalyst by reacting CH_4_ with surface oxygen species to form methyl radicals with the first C–H bond cleavage, and then it continues in a gas-phase coupling step involving the coupling of methyl radicals to C_2_H_6_ followed by dehydrogenation to C_2_H_4_^8,28^. In our work, the oxygen species are continuously produced and transported to the anode while the metal–oxide interfaces at nanoscale would be highly favorable to accommodate these active oxygen species to react with CH_4_ to initiate the first C–H bond cleavage under external applied potentials. Here the theoretical calculations are tentatively conducted to understand the cleavage of C–H bond in CH_4_ especially at the nanoscale metal–oxide interfaces. We firstly study the CH_4_ activation over the (Fe/Mo)O_2_ termination on the SFMO (001) surface as shown in Supplementary Fig. 6. In this step, a CH_4_ molecule from the feed gas dissociatively adsorbs on a metal site and a neighboring oxygen atom, forming a hydroxyl group with O on the surface and releasing a methyl radical. As shown in Fig. 3d and Supplementary Fig. 7, our calculations suggest that molecular adsorption of CH_4_ on one Fe site with the bond length of 3.263 Å is energetically unfavorable. After the C–H bond cleavage of CH_4_, the CH_3_ radical adsorbs on surface metal Fe with the bond length of 2.146 Å and the H atom binds with the O atom of SFMO surface forming O–H bond (0.987 Å). And the energy barrier of hydrogen abstraction reaction is up to 1.0 eV on the SFMO (001) surface, indicating an energetically unfavorable process for CH_4_ activation. In contrast, for the Fe/SFMO system, the CH_4_ mainly adsorbs at the interface between Fe clusters and SFMO surface. The adsorption energy of CH_4_ is −0.26 eV, where the CH_4_ binds with the Mo atom (3.734 Å) of the SFMO surface and together with the Fe atom (3.545 Å) of Fe clusters. After the first C–H bond cleavage of CH_4_, the CH_3_ radical adsorbs on the Fe atoms of Fe clusters with the bond lengths of 2.027 and 2.093 Å while the H atom binds with the O atom at the Fe/SFMO interface forming O–H bond (0.980 Å). The cleavage of C–H bond in CH_4_ at the metal–oxide Fe/SFMO (001) interface is highly favorable with the energy barrier as low as only 0.46 eV. At this stage, active oxygen at metal–oxide interfaces would be present in the form of new oxygen species (O_ads_) to initiate the cleavage of C–H bond in CH_4_. The energy barrier of oxygen transfer process through oxygen vacancy site is only 0.34 eV, as shown in Supplementary Fig. 8. Of course, the oxygen deficiency in SFMO would be favorable for CH_4_ activation and oxidation^14,29^. As we know, the charge transfer from adsorbed CH_4_ molecule to catalyst surface may be another key factor in CH_4_ activation^1^. In the anode, the adsorbed CH_4_ coupled with active oxygen species would continuously donate free electron to external circuit under electrochemical potentials and thus facilitates CH_4_ activation at the metal–oxide interfaces. It can be therefore concluded that the metal–oxide interface with active oxygen species would be effective to accommodate the reaction of C–H bond cleavage under externally applied potentials in the porous anode.

**Fig. 4:**
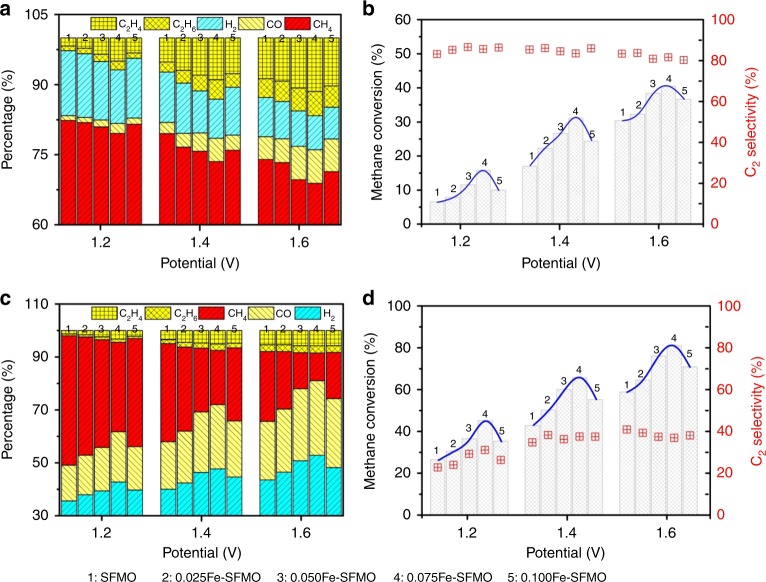
CH_4_ conversion and C_2_ selectivity in conjunction with O_2_ electrolysis. **a** The product analysis of electrochemical oxidation of CH_4_ with different anodes. **b** C_2_ selectivity and CH_4_ conversion. **c** The product analysis of electrochemical CH_4_ oxidation with composite anodes. **d** CH_4_ conversion and C_2_ selectivity after cycling the output gas

Figure 4a shows the product analysis result of electrochemical oxidation of CH_4_ with different composite anodes while the oxide ions are ionically pumped to the anodes from air in the cathode. CH_4_ with 100% concentration is supplied to the anode and the products from the anode exhaust stream are analyzed using an online gas chromatography. We firstly investigate the thermal splitting of CH_4_ to C_2_H_4_ without externally applied voltage and find that only ~0.4% C_2_H_4_/C_2_H_6_ is generated with H_2_ of around 1.6%, as shown in Supplementary Fig. 9. We also observe slight carbon deposition in this process if without electrochemical pumping oxygen ions to the anodes. When voltages are applied to the cell, it is observed that both applied voltages and metal–oxide interfaces on the anode scaffold impact the electrochemical process. C_2_H_4_ has been formed and its concentrations show a strong and positive dependence on the applied potentials, which confirms the successful conversion of CH_4_ to C_2_H_4_ using this electrochemical approach. C_2_H_6_ has also been formed through the oxidative coupling of CH_4_ in the electrochemical conversion process while its concentrations are ~1/3 of the C_2_H_4_ concentrations. The by-products are mainly H_2_ together with small amount of CO; however, their concentrations are in a close decreasing trend with the C_2_ (C_2_H_4_ + C_2_H_6_) product generations. We show that the C_2_ products reach 16.7% (11.5% C_2_H_4_ + 5.2% C_2_H_6_) with 82.2% C_2_ selectivity while the CH_4_ conversion ratio is approaching as high as 40.5% in Fig. 4b at ambient pressure and 1.6 V. This represents the highest C_2_H_4_ yields and C_2_ selectivity in the reported catalytic conversion of CH_4_ including non-oxidative and oxidative conversion processes. The C atom efficiency is reaching ~98–100% with negligible over oxidation of CH_4_ to CO_2_, which further indicates the exceptionally high efficiency for CH_4_ conversion in this electrochemical process. The SFMO itself is an oxygen-nonstoichiometric catalyst and the iron species with unsaturated oxygen coordination at the SFMO surface would deliver catalytic activity. In contrast, redox stable La_0.75_Sr_0.25_Cr_0.5_Mn_0.5_O_3−*δ*_ (LSCM) electrode without iron dopant in the lattice shows negligible catalysis activity toward C_2_H_4_ generation^11^. It is also observed that the metal–oxide interfaces enhance C_2_ product generation by ~50–100%, indicating the favorable cleavage of C–H bond at metal–oxide interfaces. Here the electrochemical processes are therefore dominated by the synergistic effect of the applied voltages and metal–oxide interfaces in the anodes. We further cycle the output gas for the electrochemical oxidation process and confirm that CH_4_ conversion has been enhanced to ~81.1% in Fig. 4c; however, the main products are syngas while the C_2_ selectivity remains ~41% (Fig. 4d). This indicates that the direct electrochemical oxidation of CH_4_ dominates the C_2_H_4_ generation, and the cycling oxidation degrades the C_2_ selectivity with the increase of syngas generation. We further conduct control experiment of electrochemical oxidation of C_2_H_6_ in the anode and we observe that the dominant products are C_2_H_4_, CH_4_, and H_2_ even without applied voltages as shown in Supplementary Fig. 10. This may be due to the thermal splitting of C_2_H_6_; however, significant coking is present in this process. When we apply voltage, the concentration of hydrocarbon product is slightly increased but the serious coking problem leads to rapid degradation of the cell performance. We therefore believe that the cycling oxidation increases syngas generation with enhanced CH_4_ conversion, while the C_2_ selectivity generally remains unchanged, indicating that the dominant C_2_ selectivity comes from the initial electrochemical oxidation process.

**Fig. 5:**
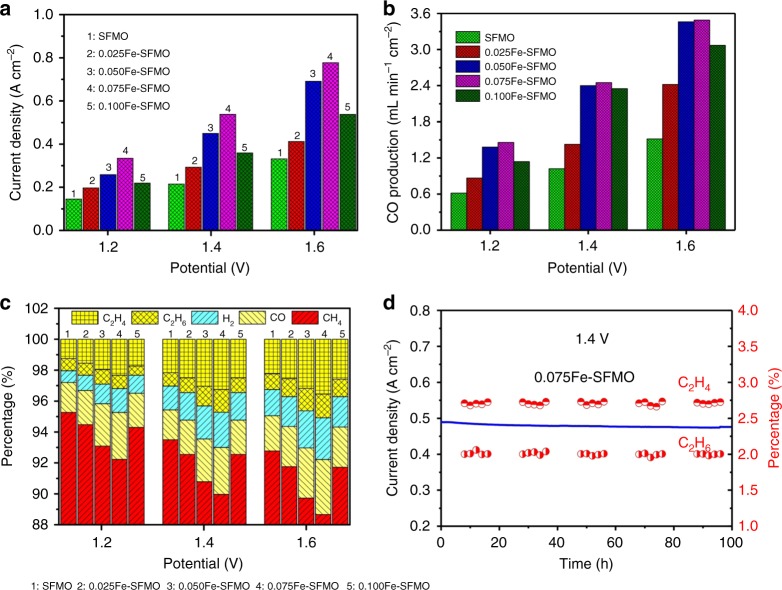
Electrochemical oxidation of CH_4_ in conjunction with CO_2_ electrolysis. **a** The current–voltage (*I–V*) relationship of CO_2_ electrolysis with CH_4_ oxidation at 850 °C. **b** CO production in the cathode at various voltages. **c** The product analysis in the anode. **d** The long-term performance of the 0.075Fe–SFMO–SDC electrode for CH_4_ oxidation with CO_2_ electrolysis at 850 °C

This simultaneous electrochemical process involves ionically pumping oxide ions from the cathode, across the electrolyte, to the anode for CH_4_ conversion. This feature provides an opportunity to convert oxygen-containing molecules, such as CO_2_ into CO while the CH_4_ conversion in the anode is maintained. Figure 5a shows the current–voltage (*I–V*) relationship of CO_2_ electrolysis in the cathode in conjunction with CH_4_ oxidation in the anode in a simultaneous process. The growth of metal–oxide interfaces in the porous electrode scaffolds also improves the current densities while the optimum composition is 0.075Fe–SFMO in which the content of metal nanoparticle dominates the metal–oxide interface length. Figure 5b gives the dependence of CO generation from CO_2_ electrolysis both on metal–oxide interfaces and applied voltages, which indicates that the higher voltages facilitate CO_2_ reduction while optimum composition is the 0.075Fe–SFMO composite with proper interface length. Figure 5c shows the CH_4_ conversion toward chemicals in the anode while the CO_2_ electrolysis is simultaneously performed in the cathode. Similar phenomenon has been observed for the performance dependence on the synergy of applied voltages and metal–oxide interfaces on the anode scaffold. The C_2_ products reach 2.04–5.08% (C_2_H_4_ + C_2_H_6_) with 75.6% C_2_ selectivity while the CH_4_ conversion ratio reaches 6.02–13.72% in the anode as shown in Fig. 5c at ambient pressure and 1.2–1.6 V. This lower performance of CO_2_/CH_4_ conversion in contrast to O_2_/CH_4_ conversion in the electrochemical process would be attributed to the higher thermodynamic and kinetic barrier of CO_2_ electrolysis than O_2_ splitting in the cathode under applied potentials. However, these performances of CH_4_ conversion and C_2_ yields/selectivity are still much higher or comparable to the reported work in catalytic CH_4_ conversion including non-oxidative and oxidative conversion processes. We calculate the current efficiency and the results are shown in Supplementary Fig. 11. The current efficiency can reach ~100% for the O_2_ electrolysis in conjunction with CH_4_ oxidation in the anode. However, the current efficiency can only reach ~80% for CO_2_ electrolysis with simultaneous CH_4_ oxidation in the anode. Electrolysis of O_2_ is highly favorable and much easier than CO_2_ electrolysis even under the similar operation conditions. We believe that the current leakage may be present in CO_2_ electrolysis in the cathode, which therefore leads to lower CH_4_ conversion under the similar conditions. The loss of current efficiency may be due to the transport of impurities including p-type hole conduction in the LSGM electrolyte, which therefore limits the transport of oxygen species and accordingly degrades the CH_4_ conversion and hydrocarbon generation.

Long-term stability of CH_4_ conversion at high temperature remains a major challenge due to the nanoparticle agglomeration and carbon deposition leading to performance degradation^8^. In this work, the iron nanoparticles are exsolved and anchored on porous SFMO scaffold through a phase decomposition process, which is expected to possess enhanced high-temperature stability against nanoparticle agglomeration. For CH_4_ conversion, carbon deposition is another challenge that leads to severe deactivation of metal nanoparticles. The carbon coking normally originates from the disproportionation of CO and the pyrolysis of CH_4_. As shown in Fig. 5d, the all ceramic symmetric cell demonstrates excellent stability even after 10 redox cycles and 100 h of operations within the frames of these experiments. The details of durability tests are shown in Supplementary Fig. 12. The generation and selectivity of C_2_ hydrocarbons remain stable even after such a tough operation at high temperatures. The exsolved metal/oxide interfaces at nanoscale are expected to improve both long-term and redox stability by preventing nanoparticle coalescence driven by surface energy reduction. One reason may be that the strong interactions at the exsolved metal–oxide interfaces produce typical interfacial charge transfer to enhance coking resistance at high temperatures. The other important reason would be that the carbon removal at the interfaces is highly favorable with electrochemically provided oxygen species in the anode because the oxygen ions are continuously transported from the cathode to the anode under externally applied voltages, which would electrochemically oxidize the deposited carbon to carbon monoxide gas, and thus substantially enhance coking resistance. We further use SEM to observe the porous electrode before and after the redox cycling experiments over 100 h. In Supplementary Fig. 13, we can see the clear microstructures of the porous electrode which well adheres to the LSGM electrolyte. After the redox cycling experiment over 100 h, we still observe the porous electrode adhered well to the LSGM electrolyte, indicative of the strong interface interaction between the electrode and the electrolyte. However, it should be noted that the iron nanoparticles generally remain unchanged while no obvious carbon deposition is observed, which again demonstrates enhanced resistance to nanoparticle agglomeration, sintering, and coking due to the anchored interface in the porous electrode.

## Discussion

In conclusion, we demonstrate a conceptually different approach of highly efficient conversion of CH_4_ to C_2_H_4_ with exceptionally high C_2_H_4_ yield and C_2_ selectivity in a solid oxide electrolyzer. The growth of metal–oxide interfaces at nanoscale with strong interactions in the porous electrode scaffolds possesses not only improved activity of CH_4_ activation but also enhanced coking resistance and thermal stability. Electrochemical oxidation of CH_4_ in the anode in conjunction with O_2_ electrolysis in the cathode shows the highest C_2_ yields of 16.7% (11.5% C_2_H_4_ + 5.2% C_2_H_6_) with 82.2% C_2_ selectivity and exceptionally high durability even after 100 h of high temperature operation. Another efficient process with high C_2_ yields has also been demonstrated with electrochemical oxidation of CH_4_ in the anode in conjunction with CO_2_ electrolysis in the cathode. The active oxygen species electrochemically pumped to the confined metal–oxide interface in the anode scaffold enable both carbon removal and CH_4_ activation. This strategy provides a reliable and efficient electrochemical process for conversion of CH_4_ into valuable chemicals. The growth of the metal–oxide interface can give rise to an interfacial interaction that facilitates catalytic conversion using CeO_2_, SrTiO_3_, TiO_2_, and MnO_2_ substrates. The catalytically active Fe or Ni can be directly doped into the lattice of the oxide substrate during materials synthesis, and then the metal nanoparticles would be grown to anchor on the oxide surface after reduction. These metal–oxide interfaces with enhanced stability and coking resistance could have broad potential applications in many other catalysis fields.

## Methods

### Synthesis

Synthesis of SFMO, 0.025Fe–SFMO, 0.050Fe–SFMO, 0.075Fe–SFMO, and 0.100Fe–SFMO (Sr_2_Fe_1.5+*x*_Mo_0.5_O_6−*δ*_; *x* = 0–0.1) were carried out by a microwave-assisted combustion method^25^. The Ce_0.8_Sm_0.2_O_2−*δ*_ (SDC) powders were synthesized using a combustion method^14^. LSGM was prepared by using a traditional solid-state reaction method^10,25^.

### Characterization

XRD (Miniflex 600, Japan) and XPS (ESCALAB 250Xi, USA) were used to analyze the phase formation of samples and elemental states before and after reduction, respectively. The oxygen nonstoichiometry of Sr_2_Fe_1.5+*x*_Mo_0.5_O_6−*δ*_ (*x* = 0–0.1) before and after reduction was determined using iodometric titration^30^. The microstructures of the samples and the exsolution of nanoparticles were analyzed using scanning electron microscopy (SEM; SU-8010, Japan) and high-resolution transmission electron microscopy (HRTEM; Tecnai F20, USA).

### Electrochemical measurements

The Sr_2_Fe_1.5+*x*_Mo_0.5_O_6−*δ*_ (*x* = 0–0.1) and SDC powders were mixed with a weight ratio of 65:35 and a suitable amount of cellulose and α-terpineol were added to prepare electrode slurry. The 1-mm-thick LSGM disks were used as the electrolyte support to assemble single cells with different electrodes (1 cm^2^) using a brush printing method followed by heat treatments at 1100 °C for 3 h in air. The silver current collector with 0.18 mm in diameter was coated on the electrode surfaces and then heat-treated in air at 550 °C for 0.5 h. For electrochemical tests of CH_4_ oxidation, the in situ AC impedance, long-term stability tests and the current density–voltage curve (*I−V* curve) were recorded using an electrochemical workstation (Zahner IM6, Germany). The CH_4_, C_2_H_4_, C_2_H_6_, CO, and H_2_ in the output gas were analyzed uisng an online gas chromatograph (GC; Shimazu 2014, Japan).

### Theoretical calculations

Spin-polarized DFT+U theory calculations were performed using a plane wave basis set Vienna Ab-initio Simulation Package (VASP) code^28,29^. The generalized gradient approach (GGA) was used including Perdew–Burke–Ernzerhof (PBE) functional to describe exchange and correlation. The interaction between core and valence electrons was described with the projector augmented wave (PAW) method. The energies and residual forces were converged to 10^–6^ eV and 0.02 eV Å^−1^, respectively. The plane wave cut-off used for energy calculations was set to 800 eV and the *U*–*J* value for Fe was set to 4.0 eV while no *U*–*J* parameter was used for Mo. The Sr_2_Fe_1.5_Mo_0.5_O_6_ cubic supercells of bulk SFMO with different distributions of Mo atoms were optimized with a 4 × 4 × 4 *k*-point grid and only the (Fe/Mo)O_2_ terminated surfaces were considered because they were expected to be more catalytically active than the SrO termination. By comparison of stability, the (Fe/Mo)O_2_ terminated surface model constructed from the plane diagonal-Mo bulk structure (*a* = *b* = *c* = 7.8717 Å, *α* = *β* = *γ* = 90°) was used to simulate the (001) surface. A *p*(2 × 2) superstructure with four layers (160 atoms) of the (001) surface of SFMO was used to simulate the periodic slab model. The vacuum region is 15 Å. The Fe segregation on the (001) surface slab of SFMO was mimicked by a cluster containing with 9 Fe atoms laying on the (001) surface of SFMO. A 2 × 2 × 1 *k*-point grid was used for Brillouin zone sampling of (001) Fe/SFMO surface system. The adsorption energy of CH_4_ with van der Waals Correction was calculated using the equation of ${E}_{{\mathrm{ads}}} = {E}_{{\mathrm{total}}} - {E}_{{\mathrm{Substrate}}} - {E}_{{\mathrm{CH}}_4}$, where ${E}_{{\mathrm{total}}}$ is the total energy of the adsorption system, ${E}_{{\mathrm{Substrate}}}\;{\mathrm{and}}\;{E}_{{\mathrm{CH}}_4}$ are the energy of the system without adsorption and the energy of the CH_4_ in gas phase, respectively^10^. The climbing-image nudged elastic band (CI-NEB) method was employed to calculate the energy barrier of oxygen vacancy transfer and the C–H bond cleavage in CH_4_ reaction processes^14^.

## Supplementary information

Supplementary Information

## Data Availability

The data supporting the findings of this study are available from the corresponding authors upon reasonable request.
