# Supplementary material for: Electrochemical conversion of methane to ethylene in a solid oxide electrolyzer
Source: Nat Commun. 2019 Mar 12;10:1173. doi: 10.1038/s41467-019-09083-3 (PMC6414614; doi:10.1038/s41467-019-09083-3)
Supplement: Supplementary file 1 — Supplementary Information [file 41467_2019_9083_MOESM1_ESM.pdf]

Supplementary Information

Electrochemical conversion of methane to ethylene in a solid oxide electrolyser

Zhu et al.

Supplementary Figures

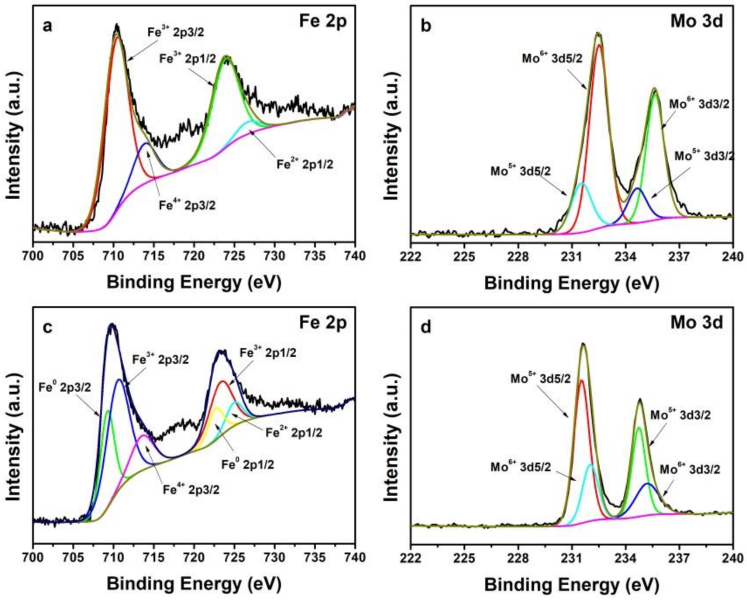

Supplementary Fig. 1. Chemical states of Fe and Mo. X-ray photoelectron spectra of Fe 2p (a) and Mo 3d (b) in the oxidized 0.075Fe-SFMO sample and Fe 2p (c) and Mo 3d (d) in reduced 0.075Fe-SFMO sample. Here the 0.075Fe-SFMO denotes the chemical formula of $Sr_2Fe_{1.575}Mo_{0.5}O_{6-\delta}$ and the reduction treatment leads to the exsolution of the metallic iron nanoparticles.

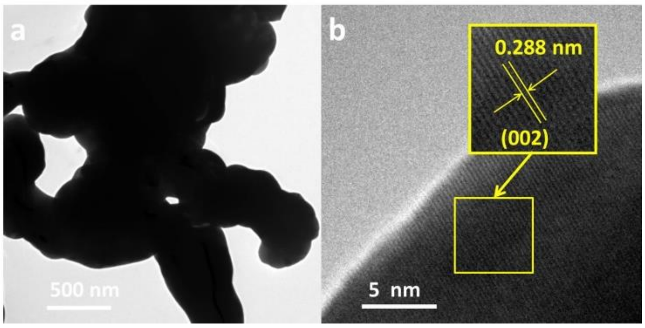

Supplementary Fig. 2. TEM results of the oxidized 0.075Fe-SFMO scaffold. The porous microstructure is shown in (a) while the lattice spacing of 0.288 nm in (b) is in accordance with (002) plane. Here the 0.075Fe-SFMO denotes the chemical formula of $Sr_2Fe_{1.575}Mo_{0.5}O_{6-\delta}$.

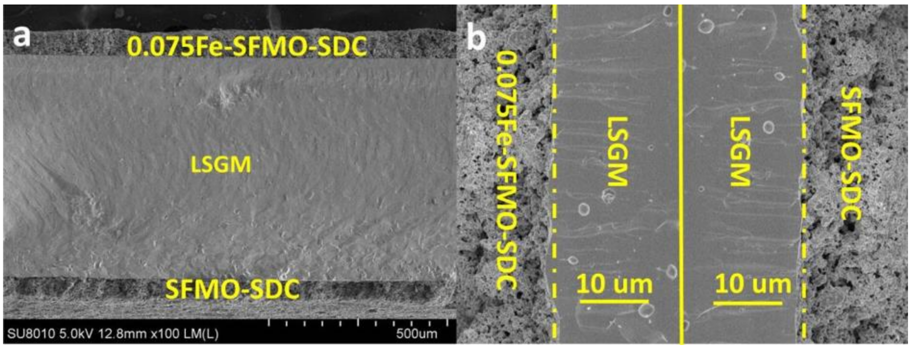

Supplementary Fig. 3. Cross-sectional SEM images for LSGM electrolyte-supported single cells with porous electrodes. Here the $Sr_2Fe_{1.5}Mo_{0.5}O_{6-\delta}$, $La_{0.9}Sr_{0.1}Ga_{0.8}Mg_{0.2}O_{3-\delta}$ and $Ce_{0.8}Sm_{0.2}O_{2-\delta}$ are denoted as SFMO, LSGM and SDC, respectively. (a) shows the cross-section of the entire cell while (b) is the enlarged view of the two electrode/electrolyte interfaces with the porous electrode and dense electrolyte.

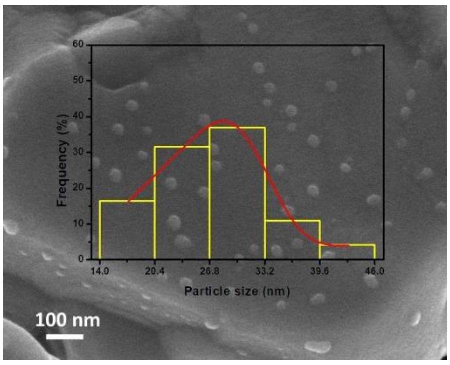

Supplementary Fig. 4. Microstructure of 0.075Fe-SFMO electrode. SEM image of the porous 0.075Fe-SFMO electrode after reduction and the iron nanoparticles have an average size of ~25 nm on the porous scaffolds. Here the 0.075Fe-SFMO denotes the $Sr_2Fe_{1.575}Mo_{0.5}O_{6-\delta}$ after reduction with iron nanoparticles anchoring on the substrate.

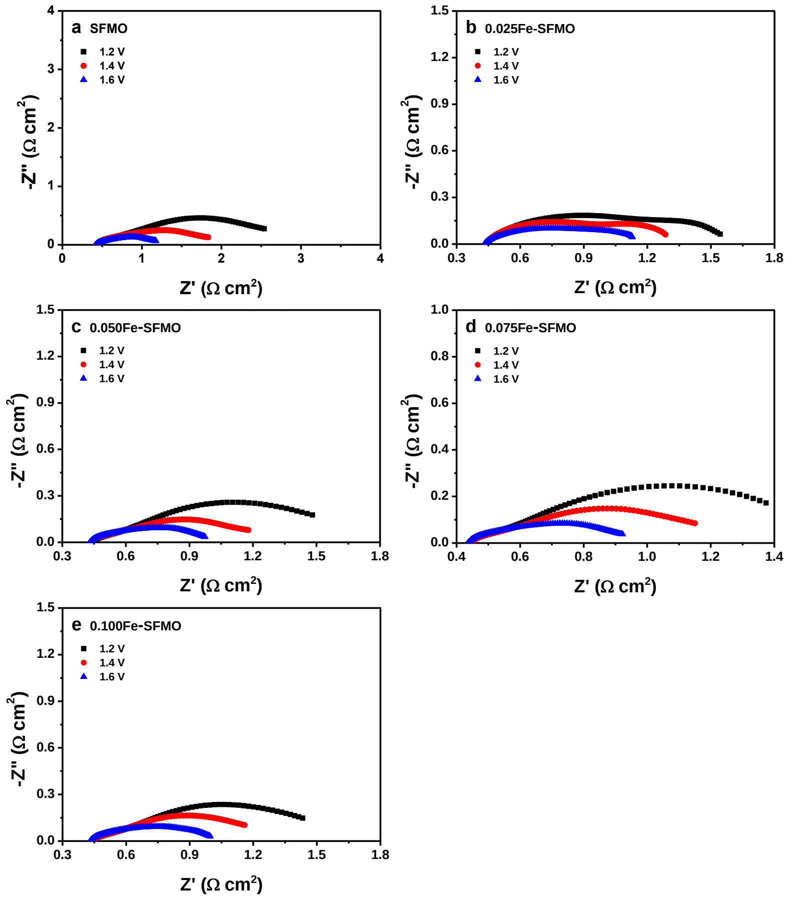

Supplementary Fig. 5. AC impedance of single cells with electrochemical oxidation of CH_4 in different anodes while the O_2 electrolysis is performed in the SFMO cathode at different applied voltages at 850 ℃. The chemical formulas of $Sr_2Fe_{1.5}Mo_{0.5}O_{6-\delta}$, $Sr_2Fe_{1.525}Mo_{0.5}O_{6-\delta}$, $Sr_2Fe_{1.55}Mo_{0.5}O_{6-\delta}$, $Sr_2Fe_{1.575}Mo_{0.5}O_{6-\delta}$ and $Sr_2Fe_{1.6}Mo_{0.5}O_{6-\delta}$ are denoted as SFMO, 0.025Fe-SFMO, 0.050Fe-SFMO, 0.075Fe-SFMO and 0.100Fe-SFMO, respectively.

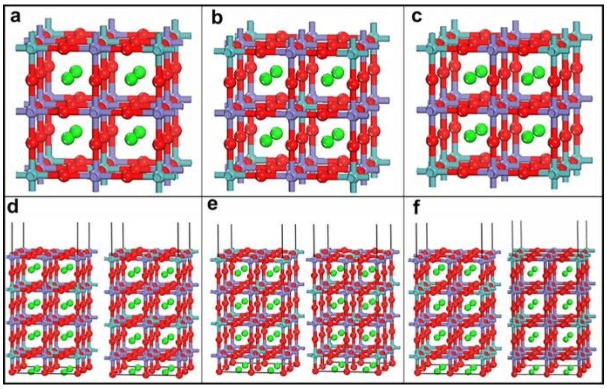

Supplementary Fig. 6. $Sr_2Fe_{1.5}Mo_{0.5}O_6$ cubic supercells of bulk SFMO with different distributions of Mo atom. (a) plane diagonal-Mo (b) body diagonal-Mo (c) plane adjacent-Mo. And (d-f) are the SFMO (001) surface models constructed of different terminated surfaces，respectively. Strontium in green, iron in purple, molybdenum in blue, oxygen in red.

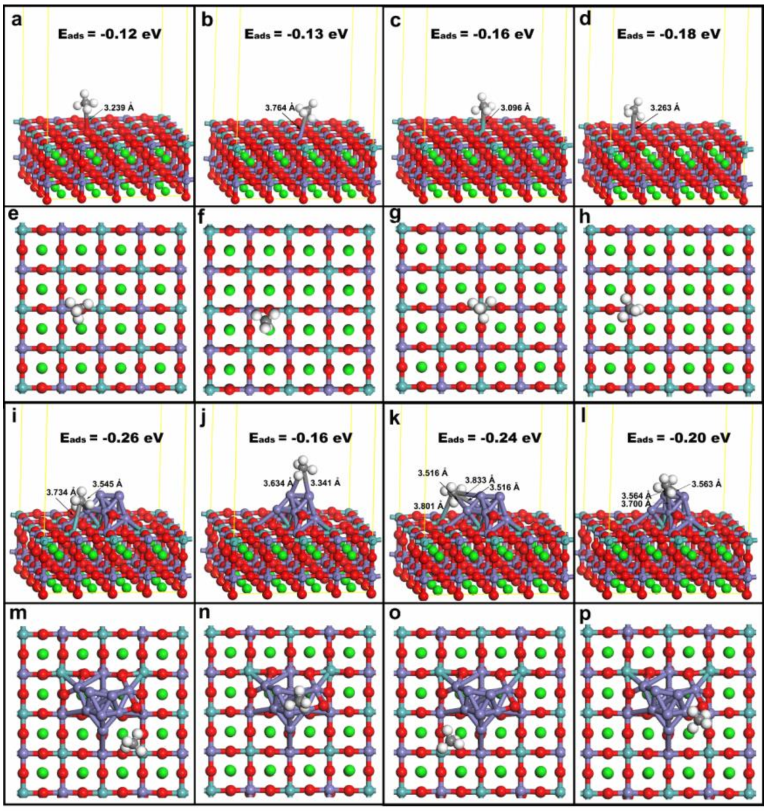

Supplementary Fig. 7. Adsorption configurations of CH_4 at surface and interface. The optimized adsorption configurations of CH_4 on (a-h) the SFMO (001) surface system and (i-p) Fe/SFMO (001) surface system. The top panels show side views while the bottom panels give top views of CH_4 adsorbed on the system. The adsorption energies of CH_4 are listed and the main bond lengths are marked. Strontium in green, iron in purple, molybdenum in blue, oxygen in red, carbon in grey and hydrogen in white.

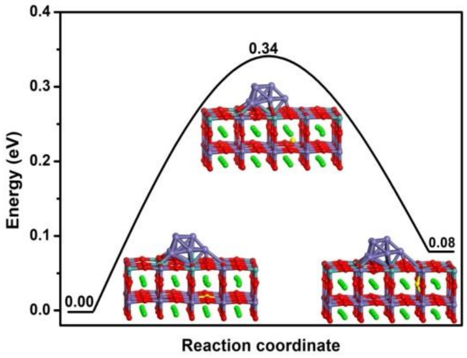

Supplementary Fig. 8. The activation energy barrier of oxygen transfer process through oxygen vacancy sites. Strontium in green, iron in purple, molybdenum in blue, oxygen in red and yellow.

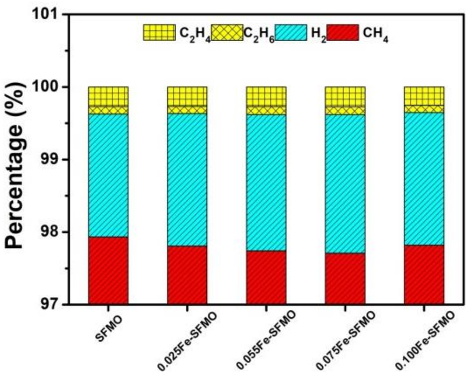

Supplementary Fig. 9. Contrast experiment of ethylene splitting. The ethylene generation from thermal splitting methane in the cell without externally applied voltages at operation temperature of 850 ^oC.

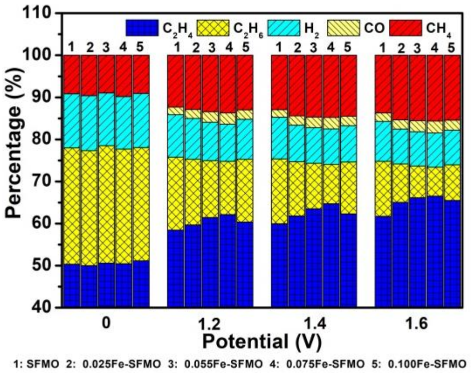

Supplementary Fig. 10. Electrochemical oxidation of C_2H_6. The product analysis of electrochemical oxidation of C_2H_6 in the anode in conjunction with O_2 electrolysis from air in the cathode at ambient pressure and 850°C.

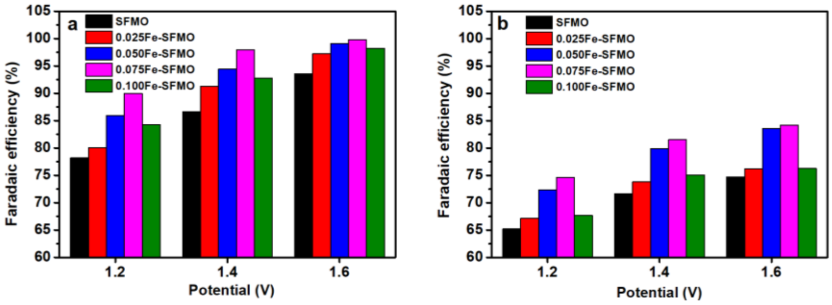

Supplementary Fig. 11. Faraday efficiency of electrochemical process. Faraday efficiency of CH_4 oxidation with (a) O_2 electroylysis and (b) CO_2 electrolysis using various cathodes and different voltages at 850 ℃.

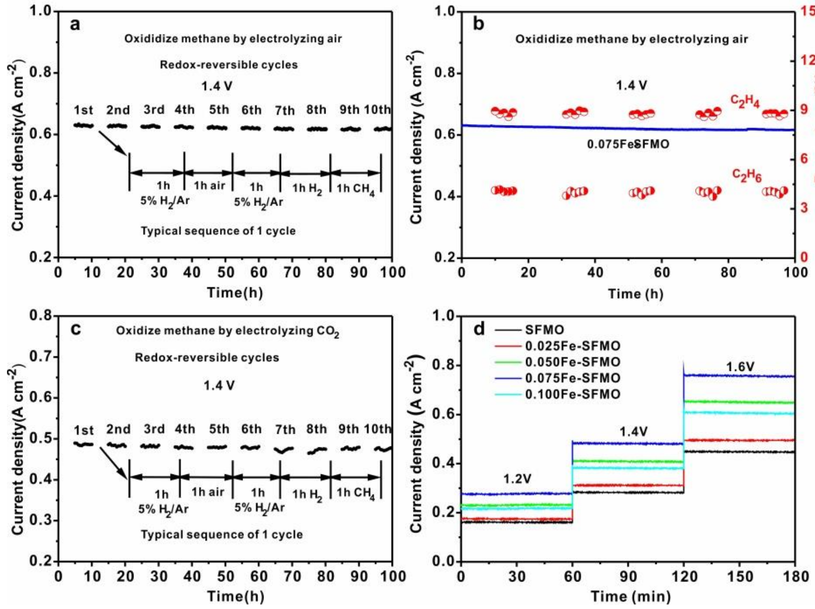

Supplementary Fig. 12. Electrochemical measurement of single cells. (a) The redox-cycling performance of the cell with 0.075Fe-SFMO-SDC anode for oxidizing methane by electrolyzing air at 850 ℃. (b) The long-term performance of the cell with 0.075Fe-SFMO-SDC anode for oxidizing methane by electrolyzing air with the percentage of CH_4 conversion to C_2H_4 and C_2H_6 at 850 ℃. (c) The redox-cycling performance of the cell with 0.075Fe-SFMO-SDC anode for oxidizing methane by electrolyzing CO_2 at 850 ℃. (d) The short-term operation of oxidizing methane by electrolyzing CO_2 at different voltages.

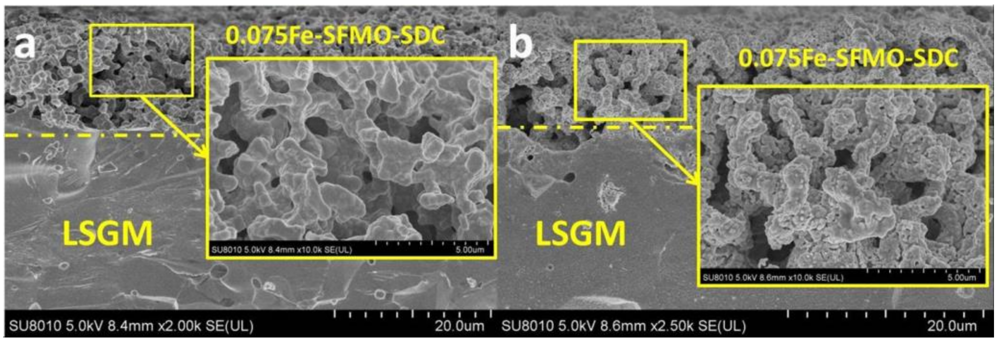

Supplementary Fig. 13. SEM images of the 0.075Fe-SFM/SDC electrodes. (a) represents the porous electrode before reduction while (b) is the microstructure after the redox cycling experiment over 100 hours test.

Supplementary Tables

Supplementary Table 1. Oxygen nonstoichiometry of samples before and after nanoparticle exsolution. The oxygen is determined using iodometric titration while the amounts of excess iron in the lattice of SFMO are in a linear relationship with the further oxygen loss after reduction.

Samples	Chemical formula	Oxidized state (6-δ)	Reduced state (6-δ)	Oxygen loss
SFMO	$Sr_2Fe_{1.5}Mo_{0.5}O_{6-\delta}$	5.6616	5.4151	0.2465
0.025Fe-SFMO	$Sr_2Fe_{1.525}Mo_{0.5}O_{6-\delta}$	5.6940	5.3963	0.2977
0.05Fe-SFMO	$Sr_2Fe_{1.55}Mo_{0.5}O_{6-\delta}$	5.7344	5.3693	0.3651
0.075Fe-SFMO	$Sr_2Fe_{1.575}Mo_{0.5}O_{6-\delta}$	5.7664	5.3771	0.3893
0.100Fe-SFMO	$Sr_2Fe_{1.6}Mo_{0.5}O_{6-\delta}$	5.7838	5.4732	0.3105
